# An Efficient *Agrobacterium*-Mediated Transformation System for Poplar

**DOI:** 10.3390/ijms150610780

**Published:** 2014-06-13

**Authors:** Ali Movahedi, Jiaxin Zhang, Rasoul Amirian, Qiang Zhuge

**Affiliations:** 1 The Cooperative Innovation Center of Southern Modern Forestry, Nanjing Forestry University, Nanjing 210037, China; E-Mails: ali664699@gmail.com (A.M.); zhangjiaxin60633@163.com (J.Z.); 2 Department of Genomics, Agricultural Biotechnology Research Institute, Central Region of Iran (ABRICI), Najafabad Road, Isfahan 85135-487, Iran; E-Mail: rasool_amirian@yahoo.com

**Keywords:** transformation system, *Populus*, *Agrobacterium*-mediated, efficient

## Abstract

Poplar is a model system for the regeneration and genetic transformation of woody plants. To shorten the time required for studies of transgenic poplar, efforts have been made to optimize transformation methods that use *Agrobacterium* *tumefaciens*. In this study, an *Agrobacterium* infective suspension was treated at 4 °C for at least 10 h before infecting explants. By transforming the *Populus* hybrid clone “Nanlin895” (*Populus deltoides* × *P. euramericana*) with *Agrobacterium* harboring the *PBI121:CarNAC6* binary vector, we showed that the transformation efficiency was improved significantly by multiple independent factors, including an *Agrobacterium* infective suspension with an OD_600_ of 0.7, an *Agrobacterium* infection for 120 min, an *Agrobacterium* infective suspension at a pH of 5.0, an acetosyringone concentration of 200 µM, a cocultivation at 28 °C, a cocultivation for 72 h and a sucrose concentration of 30 g/L in the cocultivation medium. We also showed that preculture of wounded leaf explants for two days increased the regeneration rate. The integration of the desired gene into transgenic poplars was detected using selective medium containing kanamycin, followed by southern blot analysis. The expression of the transgene in the transgenic lines was confirmed by northern blot analysis.

## 1. Introduction

Poplar is a versatile tree species that is highly amenable to vegetative propagation, has a rapid growth rate and is a good model system for the transformation of woody plant species. These characteristics have led to its widespread use in the pulp, paper and cosmetics industries and in both highland and lowland reforestation [1,2,3,4]. Furthermore, poplar is a forest tree species, with a rather long generation cycle (seven to 10 years or more). That is why researchers usually use materials from the initial generation for the analysis of transgenic plants of forest trees, with just several months’ generation cycles. Efforts to optimize regeneration and propagation methods that use leaves, petioles, internodes, stems, roots and sprouts to improve poplar through the overexpression of transgenes conferring resistance to biotic and abiotic stresses or other traits have increased markedly over the past decade [2,3,5,6]. Transformation methods using leaf segments are the most commonly used [7]. Attempts to improve poplar transformation mediated by *Agrobacterium tumefaciens* have focused on a variety of parameters, including different *Agrobacterium* strains and culture densities, various acetosyringone (AS) concentrations and other factors[1,8,9,10,11].

The expression of high-copy number transgenes in transgenic plants may be more or less than only a single copy of transgene, and also, sometimes, this is good for molecular analysis and genetic engineering. Furthermore, Husaini [12] reported that strawberry transgenic plants with a high transgene copy number (four copies or more) are very good for molecular analysis and genetic engineering. Moreover, Bartlett *et al.* [13] reported that an improved method in barely using *Agrobacterium*, with a high copy of transgenes, is quite good for molecular analysis and genetic engineering. The benefits of improved transformation methods include the insertion of intact full-length cDNAs into the plant genome, a reduction in the number of undesirable mutations and an increased transgene copy number [4]. For these reasons, many efforts have been made to improve *Agrobacterium*-mediated transformation efficiency [14,15]. The *CarNAC6* gene from chickpea encodes a 308-amino-acid, plant-specific regulatory transcriptional factor belonging to the NAC (*NAM*, *ATAF1,2*, and *CUC2*) domain protein family that increases plant response reactions to biotic and abiotic stresses and plays an important role in plant development. Homologues of the *CarNAC6* gene have been identified in *Arabidopsis* and rice [16,17,18,19,20]. In this study, we transformed the poplar hybrid clone “Nanlin895” (*Populus deltoides* × *P. euramericana* “Nanlin895”) with the *PBI121:CarNAC6* binary vector to improve *Agrobacterium*-mediated transformation efficiency. In addition, we attempted to optimize MS culture medium supplemented with *N*-6-benzyladenine (6-BA) and thidiazuron (TDZ) hormones to minimize lateral shoot development. Other factors affecting transformation efficiency evaluated in this study included the preculture of wounded explants, *Agrobacterium* infective suspension concentration, *Agrobacterium* infection duration, *Agrobacterium* infective suspension pH, acetosyringone (AS) concentration, cold treatment of an *Agrobacterium* infective suspension, cocultivation incubation temperature and duration and sucrose concentration in cocultivation medium. All of these factors were treated independently in related experiments independent of the age of the explants. In this study, the transformation efficiency is expressed as the percentage of independently transformed explants relative to the total number of explants.

## 2. Results and Discussion

### 2.1. Optimization of Poplar Regeneration

**Figure 1:**
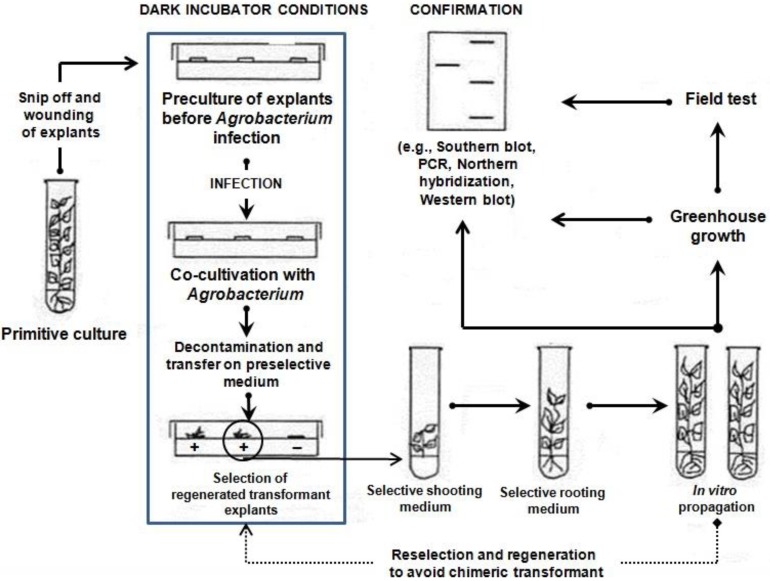
An overview the steps for *Agrobacterium*-mediated transformation in *Populus* species.

**Figure 2:**
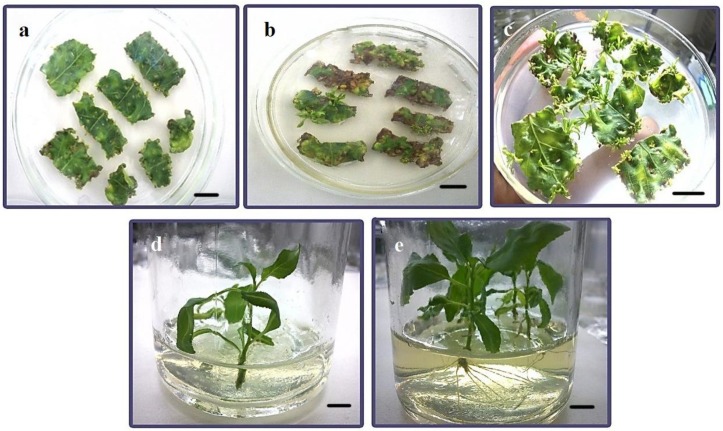
(**a**) Cocultivation of poplar leaf discs with *Agrobacterium* on MS medium; (**b**) Regenerated putative poplar transformants on MS selection medium that were generated without the optimization of transformation efficiency; (**c**) Regenerated putative poplar transformants on MS selection medium generated with the optimization of transformation efficiency; (**d**) MS shoot elongation medium; (**e**) Half MS rooting medium. Scale bar = 1 cm.

**Figure 3:**
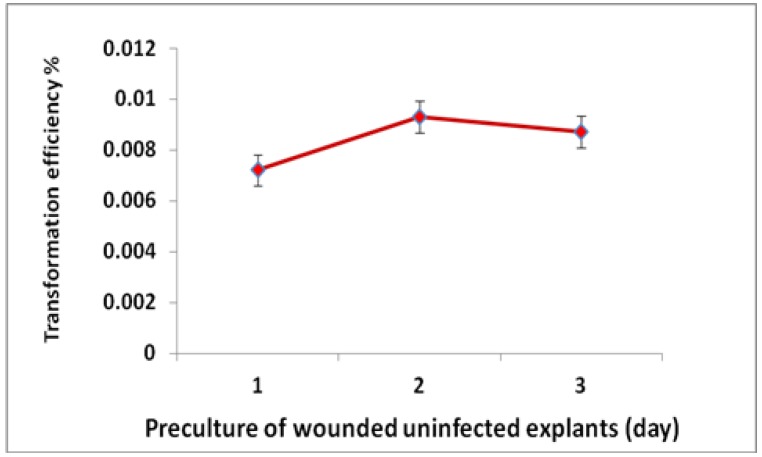
Precultured, wounded poplar explants before induction by *Agrobacterium*.

An overview of the steps used in the *Agrobacterium*-mediated transformation of *Populus* species is presented in Figure 1. The poplar hybrid clone “Nanlin895” (*Populus deltoides × P. euramericana* “Nanlin895”) was cultured in medium containing various concentrations of 6-BA and TDZ (Table 1). Little or no regeneration occurs in the absence of 6-BA. Medium containing 0.5 mg/L 6-BA was used to achieve the maximum regeneration rate (85% to 90%). Increases in non-specific poplar regeneration resulted in higher numbers of lateral shoots relative to the numbers of main shoots. TDZ, an inducer of shoot regeneration, was used to increase the specific regeneration of main shoots. The proportion of main shoots to lateral shoots was increased by TDZ concentrations of 0.002 and 0.004 mg/L, but was decreased by concentrations of 0.008 and 1.0 mg/L. The optimal regeneration medium contained 0.5 mg/L 6-BA and 0.004 mg/L TDZ to maximize the frequency of main shoot regeneration. The rates of shoot elongation (Figure 2d) and root regeneration (Figure 2e) were enhanced by up to 100% when using the optimized medium. As shown in Figure 2a and Figure 3, the preculture of explants for two days caused higher transformation efficiencies than preculture for one and three days. Therefore, explants precultured for two days were used subsequently for transformation with *Agrobacterium.*

**Table 1:** The effect of *N*-6-benzyladenine (6-BA) and thidiazuron (TDZ) on the main shoot and total shoot regeneration (%).

Medium Number	6-BA (mg/L)	TDZ (mg/L)	Mean Number of Main Shoots per Medium (Number of Main Shoots in Each Medium/Number of Explants in Each Medium)	Total Shoot Regeneration in Each Medium (%)	
1	1.00	0.000	3.10	89.0	
2	1.00	0.002	3.50	93.0	
3	1.00	0.004	4.80	94.0	
4	1.00	0.008	3.30	89.0	
5	1.00	0.010	2.90	97.0	
6	0.80	0.000	38.8	85.0	
7	0.80	0.002	43.0	82.0	
8	0.80	0.004	45.8	78.0	
9	0.80	0.008	40.7	80.0	
10	0.80	0.010	30.8	79.0	
11	0.50	0.000	56.8	80.0	
12	0.50	0.002	65.2	75.0	
13	0.50	0.004	69.5	73.0	
14	0.50	0.008	60.5	76.0	
15	0.50	0.010	44.4	70.0	
16	0.20	0.000	8.90	37.0	
17	0.20	0.002	11.0	29.0	
18	0.20	0.004	14.0	35.0	
19	0.20	0.008	9.00	32.0	
20	0.20	0.010	6.13	26.0	
21	0.00	0.000	0.30	6.00	
22	0.00	0.002	0.50	9.00	
23	0.00	0.004	0.80	13.0	
24	0.00	0.008	0.50	10.0	
25	0.00	0.010	0.10	8.00	

### 2.2. Improvement of Agrobacterium-Mediated Transformation Efficiency

**Figure 4:**
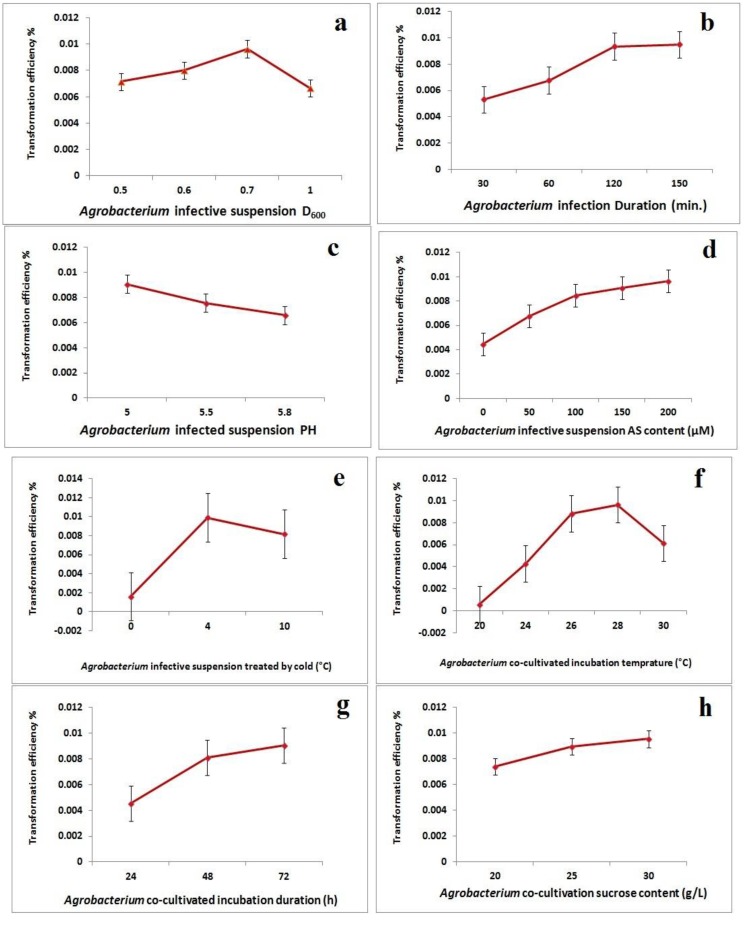
The effects of various factors on *Agrobacterium*-mediated transformation efficiency: (**a**) *Agrobacterium* concentration; (**b**) *Agrobacterium* infection duration; (**c**) *Agrobacterium* suspension pH; (**d**) acetosyringone (AS) concentration; (**e**) cold pre-treatment; (**f**) cocultivation temperature; (**g**) cocultivation duration; (**h**) cocultivation medium sucrose concentration.

The highest transformation efficiency was obtained from precultured explants immersed in an *Agrobacterium* infective suspension with an OD_600_ of 0.7. The use of *Agrobacterium* infective suspensions with higher densities resulted in decreased transformation efficiencies, due to increased infection (Figure 4a). We examined the effect of the duration of immersion in *Agrobacterium* infective suspensions on the transformation efficiency. The highest transformation efficiencies were obtained from immersion for 120 and 150 min and were not significantly different (Figure 4b). In addition, we examined the effect of the pH of the *Agrobacterium* infective suspension on the transformation efficiency. Low pHs increased the efficiency, but higher pHs decreased the transformation efficiency, due to decreased *Agrobacterium* growth (Figure 4c). Increasing the AS concentration in the *Agrobacterium* infective suspension to as high as 200 µM improved the transformation efficiency, probably by increasing the ability of the *Agrobacterium* to detect wounded plant cells. The transformation efficiencies obtained with the different AS concentrations were significantly different (Figure 4d).

We developed a cold pre-treatment of the *Agrobacterium* infective suspension inspired by the freeze-thaw method [21]. The transformation efficiency was increased approximately 7% by pre-treatment of the suspension at 4 °C for at least 24 h (Figure 4e). The effect of the cold pre-treatment of the *Agrobacterium* infective suspension on the transformation efficiency was clearly demonstrated by the differences between cultures on plates for which the suspension had not received (Figure 2b) or had received (Figure 2c) the cold pre-treatment. The temperature at which the *Agrobacterium* infective suspension and the explants were co-cultivated also affected the transformation efficiency. Cocultivation at 28 °C produced the highest transformation efficiency, with significant differences in efficiency between the temperatures tested (Figure 4f). The duration of the cocultivation of the *Agrobacterium* infective suspension and explants was an important factor in the transformation efficiency. Cocultivation for up to 72 h yielded the highest transformation efficiency, with significant differences between incubation temperatures (Figure 4g)

Sucrose is a required carbon source for the growth of *Agrobacterium*. The effects of various sucrose concentrations on transformation efficiency during the cocultivation of the *Agrobacterium* infective suspension and explants were significantly different, with the highest transformation efficiency obtained using a sucrose concentration of 30 g/L (Figure 4h).

### 2.3. Southern and Northern Blot Analyses

**Figure 5:**
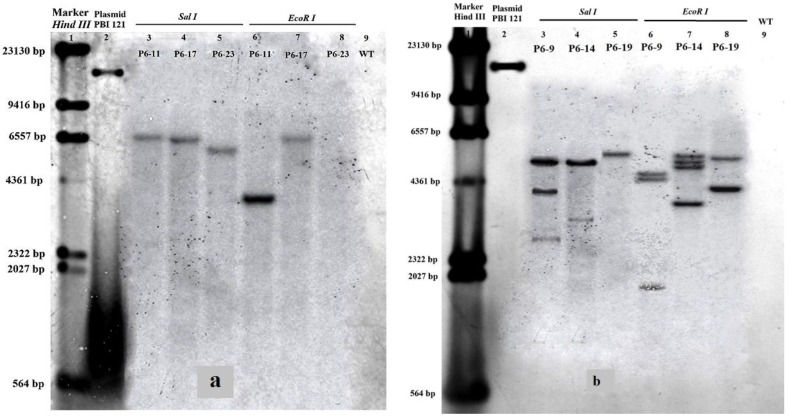
(**a**) Southern blot analysis of transgenic poplar lines generated without improved transformation efficiency. Genomic DNA extracted from three transgenic poplar lines was digested separately with *Sal*I and *Eco*RI and blotted onto a nylon membrane. The blots were probed with an 868-bp PCR fragment generated from *CarNAC6* cDNA. Lane 1: marker (ʎ *Hin*dIII). Lane 2: *PBI121:CarNAC6* plasmid digested with *Eco*RI; Lanes 3–5: genomic DNA extracted from three transgenic poplar lines digested with *Sal*I; Lanes 6–8: the same genomic DNA digested with *Eco*RI; Lane 9: wild-type (WT) poplar; (**b**) Southern blot analysis of transgenic poplar lines generated with improved transformation efficiency.

Total genomic DNA isolated from transgenic plants was digested with *Sal*I, which has no restriction site in the T-DNA, and with *Eco*RI, for which one restriction site is located downstream of the *NOS* (nopaline synthase) terminator. Southern blot analysis showed that 1–2 transgene copies were present in each of the three lines generated in procedures that did not show improved transformation efficiency (Figure 5a) and that 3–4 transgene copies were present in each of the three lines generated using procedures resulting in improved transformation efficiency (Figure 5b).

**Figure 6:**
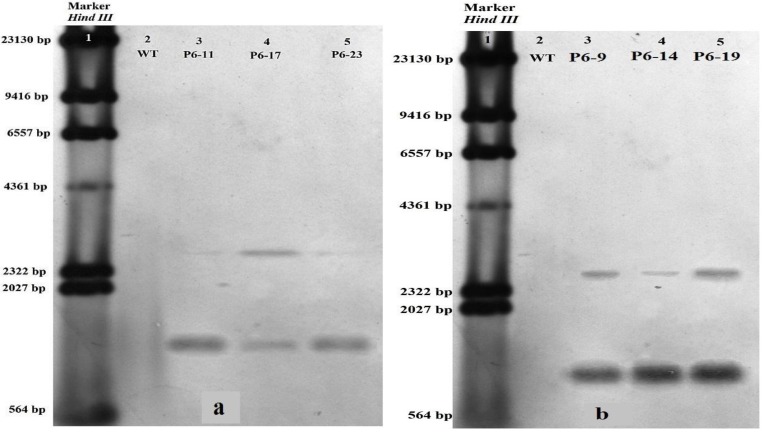
(**a**) Northern blot analysis of transgenic poplar lines generated without improved transformation efficiency. Total RNA (ribonucleic acid) was separated on a formaldehyde gel and blotted onto a nylon membrane after staining with ethidium bromide. A 433-bp fragment of *CarNAC6* cDNA generated by PCR (polymerase chain reaction) was used as a probe. *CarNAC6* gene expression (0.5–0.8-kb band) in transgenic poplar lines. Lane 1: marker (λ *Hin*dIII); Lane 2: wild-type (WT) poplar; Lanes 3–5: total RNA extracted from three transgenic poplar lines; (**b**) Northern blot analysis of transgenic poplar lines generated with improved transformation efficiency.

The detection of mRNAs of 0.5–0.8 kbp by northern blot analysis confirmed that the *CarNAC6* transgene was expressed in three transgenic poplar lines generated without improved transformation efficiency (Figure 6a) and in three transgenic poplar lines generated with improved transformation efficiency (Figure 6b). The transgene expression levels in the two line types were clearly different. Supplementation of media with hormones has been investigated to determine optimal conditions for shoot regeneration with a minimum of lateral shoots and for root regeneration. High concentrations of TDZ can result in the regeneration of a high percentage of adventitious shoots [22]. Ferreira *et al.* [2] reported that the regeneration of adventitious shoots from organogenic nodules of *P. euphratica* leaf explants was achieved using a range of α-naphthaleneacetic acid (NAA) and 6-BA concentrations. Various concentrations of indoleacetic acid (IAA) were also shown to affect the frequency of regeneration from poplar explants [23]. Confalonieri *et al.* [1] reported positive effects of BA and NAA on shoot regeneration in *P. nigra*. Various media are required for shoot and root regeneration of different genotypes and are supplemented with various levels of indole-3-butyric acid (IBA) to induce rooting from aspen leaves and with zeatin to induce shooting [24]. The use of specific concentrations of shoot-inducing hormones, such as 6-BA, can increase the mean number of shoots formed per explant following regeneration, but typically causes decreased numbers of main shoots following regeneration [4]. Recently, the number of reports of successful *Agrobacterium*-mediated transformation of various plant species has increased markedly [22]. Developments in tissue culture methods and improved transformation techniques have led to the use of suitable *Agrobacterium* strains [25]. The development of techniques that increase transformation efficiency or the pathogenicity of *Agrobacterium* has been the subject of many studies. The use of appropriate AS concentrations during cocultivation has been shown to increase transformation efficiencies. Moreover, the effects of the *Agrobacterium* concentration and infection time on transformation efficiency have been demonstrated by Han *et al.* [4].

## 3. Experimental Section

### 3.1. Plant Materials and Agrobacterium Strain

Wild-type (WT) poplar leaves were collected from young *P. deltoides × P. euramericana* “Nanlin895” seedlings to propagate 25 lines. The binary vector, *PBI121:CARNAC6*, was generated at the State Key Laboratory of Crop Genetics and Germplasm Enhancement of the National Center for Soybean Improvement at Nanjing Agricultural University, China [26]. The pBI121 plasmid was digested with *Xba*I and *Bam*HI restriction enzymes, and a 924-bp nucleotide DNA fragment containing a full-length cDNA of the chickpea *CARNAC6* gene was inserted. *CARNAC6* expression was driven by the CaMV *35S* promoter. The plasmid was introduced into the *Agrobacterium* *tumefaciens* strain, EHA105, using the freeze-thaw method [21].

### 3.2. Optimization of Regeneration Medium for Poplar

Murashige and Skoog (MS) medium [27] was supplemented with 6-benzylaminopurine (6-BA) at five concentrations (1.0, 0.8, 0.5, 0.2 and 0 mg/L) in combination with thidiazuron (TDZ) at five concentrations (0, 0.002, 0.004, 0.008 and 0.01 mg/L) to determine the optimal combination for minimal lateral shoot development. Immature leaves were excised as 1-cm^2^ discs and cultured on MS medium supplemented with the two hormones. The medium was refreshed at 2-week intervals. Regenerated shoots of 2 cm in length were cut and rooted on sterile half-strength MS medium containing 25 g/L sucrose and 6 g/L agar adjusted to pH 5.8 with no added hormones. After 6–8 weeks, the roots were examined, and leaves were collected from well-developed plantlets for gene transformation.

### 3.3. Agrobacterium-Mediated Transformation

Young leaves were cut into 0.5 × 0.5-cm sections and precultured on half-strength MS medium supplemented with 6-BA (0.5 mg/L) and TDZ (0.004 mg/L) for 1, 2 or 3 days. This medium was tested on 37–40 lines for each variable, with four replicates. *Agrobacterium* containing the binary vector, *PBI121:CARNAC6*, was inoculated into Luria–Bertani (LB) medium supplemented with 50 mg/L rifampicin (Rif) and 50 mg/L kanamycin (Kan) and cultured in an incubator without light at 28 °C for 72 h [28].

A single *Agrobacterium* colony was inoculated into 5 mL of LB medium containing 50 mg/L Rif and 50 mg/L Kan and incubated at 28 °C for 24 h with constant agitation (220 rpm). An additional 50 mL of LB were added and growth was continued overnight under the same conditions until an OD_600_ of 2.0 was reached. The culture was centrifuged at 3000 rpm for 10 min. The pellet was diluted in liquid MS medium with 5% sucrose to prepare an *Agrobacterium*-infective suspension. All *Agrobacterium* experiments were carried out independently using 20–25 precultured explants in triplicate.

The precultured explants were immersed in various concentrations of *Agrobacterium*-infective suspensions (OD_600_ values of 0.5, 0.6, 0.7 or 1.0). The precultured explants were also cold treated independently at 0, 4 or 10 °C. Other parameters tested independently for their effects on transformation efficiency included the duration of the cocultivation of the *Agrobacterium*-infective suspension with explants (30, 60, 120 or 150 min at 28 °C with constant agitation), the AS concentration (0, 50, 100, 150 or 200 µM) and the pH of the *Agrobacterium* infection suspension (pH 5.0, 5.5 or 5.8). Wounded leaf discs were dried on sterile paper to remove surface water. The infected explants were co-cultivated in a dark incubator at various temperatures (20, 24, 26, 28 or 30 °C) and for different durations (24, 48 or 72 h) on semi-liquid MS induction medium containing 0.5 mg/L 6-BA, 0.004 mg/L TDZ, 3 g/L agar, various sucrose concentrations (20, 25 or 30 g/L) and 200 µM AS to induce the expression of the *Agrobacterium* *Vir* genes [29]. The parameters used for control transformations were the preculture of explants for 1 day, an *Agrobacterium* culture OD_600_ of 0.5, 10 °C cold pre-treatment, 30 min of *Agrobacterium* cocultivation, 100 µM AS, an *Agrobacterium*-infective suspension at pH 5.8, cocultivation in darkness at 28 °C for 48 h and 25 g/L sucrose in MS induction medium. Putative transformed explants were transferred to MS selection medium supplemented with 0.5 mg/L 6-BA, 0.004 mg/L TDZ, 6 g/L agar, 25 g/L sucrose, 400 mg/L cefotaxime (Cef) and 50 mg/L kanamycin (Kan) at pH 5.8 and cultured under 16-h/8-h light/dark conditions at 23 ± 1 °C in a phytotron. Selected regenerated shoots were separated and transferred to MS shoot elongation medium supplemented with 0.25 mg/L 6-BA, 0.002 mg/L TDZ, 6 mg/L agar, 25 mg/L sucrose and 300 mg/L Cef. Shoots of approximately 2 cm in length were then transferred onto half-strength MS rooting medium supplemented with 6 mg/L agar, 25 mg/L sucrose and 300 mg/L Cef with no added hormones. Plants that were well developed were transferred to a greenhouse for experiments. Transformation efficiencies were calculated using the following equation: (number of transformed explants/total number of explants) × 100.

### 3.4. Southern and Northern Blot Analyses

For southern blot analysis, 10 µg of genomic DNA were extracted using the CTAB (cetyl trimethylammonium bromide) method [30,31] from WT plants, three transgenic poplar lines generated using procedures with no improvements to the transformation efficiency (P6-11, P6-17 and P6-23) and three transgenic poplar lines using procedures generated with improvements to transformation efficiency (P6-9, P6-14 and P6-19). The genomic DNA was digested with *Sal*I and *Eco*RI at 37 °C for 4 h and separated overnight on a 0.8% agarose gel at 15 V. The digested genomic DNA was transferred to a Hybond N+ nylon membrane (Amersham Biosciences BV, Eindhoven, The Netherlands) and detected using DIG (digoxigenin) reagent, according to the manufacturer’s instructions (catalog number 11745832910; Roche, Basel, Switzerland). The primers, F5'-TACCGAGGATATTACACTACCAGGA-3' and R 5'-AGTCCAGTTTTGCAGCCAAG-3', were used to generate an 868-bp PCR product to use as a probe on the southern blot. Northern blot analysis was performed using 7 µg of total RNA from young leaves of WT plants and three transgenic poplar lines extracted using TRIzol reagent (Tiangen Biotech, Beijing, China), according to the manufacturer’s instructions. Total RNA was separated on a 1.2% agarose formaldehyde gel, transferred to a Hybond N+ nylon membrane (Amersham Biosciences BV), and processed for northern hybridization. Random primers labelled with digoxigenin-11-dUTP using DIG-High Prime (Roche) were used to generate probes from the *CarNAC6* cDNA (GenBank Accession Number FJ477887.1). The primers, F 5'-CTGGCAAAGGTTGGAGAAAG-3' and R 5'-CGTGTTGGTCTTGTTGTTGT-3', were used to generate a 433-bp PCR product to use as a probe for northern blotting.

### 3.5. Statistical Analysis

All data analyses were performed using ANOVA (analysis of variance) with a mean separation calculated using Duncan’s test with SPSS version 16 (SPSS Inc., Chicago, IL, USA) and Excel 2013 software (Microsoft, Redmond, WA, USA). Confidence intervals showing no overlap of the mean values with an error value of 0.05 were considered to indicate statistically significant differences.

## 4. Conclusions

In this study, we demonstrated that the regeneration efficiency of hybrid poplar “Nanlin895” (*Populus deltoides* × *P. euramericana* “Nanlin895”) could be optimized to yield an 85%–90% regeneration rate. We successfully transformed poplar with the *CarNAC6* biotic and abiotic stress resistance gene using *Agrobacterium*-mediated transformation. We showed that the transformation efficiency was increased by using a two-day preculture of wounded regeneration explants, an *Agrobacterium*-infective suspension with an OD_600_ of 0.7, cocultivation of the *Agrobacterium* infection suspension with explants for 120 min, an *Agrobacterium*-infective suspension pH of 5.0, an AS concentration of 200 µM, a 4 °C cold treatment of the *Agrobacterium*-infective suspension, a cocultivation incubation temperature of 28 °C, a cocultivation duration of 72 h and a cocultivation medium sucrose concentration of 30 g/L.

## Abbreviations

6-BA: *N*-6-benzyladenine
AS: acetosyringone
*NPTII*: neomycin phosphotransferase
ORF: open reading frame
TDZ: thidiazuron
Cef: cefotaxime sodium
Kan: kanamycin sulfate
MS: Murashige and Skoog
Rif: rifampicin
NAA: naphthaleneacetic acid
IAA: indoleacetic acid
IBA: indole-3-butyric acid
*NAM*: no apical meristem
*ATAF1,2*: *Arabidopsis* transcription activation factor
*CUC2*: cup-shaped cotyledon
